# Supplementary material for: Patterns of Intron Gain and Loss in Fungi
Source: PLoS Biol. 2004 Nov 30;2(12):e422. doi: 10.1371/journal.pbio.0020422 (PMC532390; doi:10.1371/journal.pbio.0020422)
Supplement: Table S1 — Also available at http://genes.mit.edu/NielsenEtAl/. (4.3 MB ZIP). [file pbio.0020422.st001.zip › NielsenEtAl/html/1326.html]

AN5731.1.NCU05420.1.MG03281.1.FG01643.1


```
 CLUSTAL W (1.82) Multiple Sequence Alignments - Introns Inserted


Sequence 1: NCU05420.1	432 aa
Sequence 2: MG03281.1	424 aa
Sequence 3: AN5731.1	410 aa
Sequence 4: FG01643.1	395 aa
Alignment Length: 432 aa
Number Identitical Residues: 236 aa
Alignment Score (without introns) 11027


MG03281.1 	MSTFGTTFRVTT~YGESHCRSVGCIVDGCPPGMQLTEADIQPHMNRRRPGQSDITTPRNE
NCU05420.1	MSTFGHYFRVTT2YGESHCKSVGCIVDGVPPGMELTEDDIQPQMTRRRPGQSAITTPRDE
FG01643.1 	MSSFGQFFRVTT2AGESHGKSVSCIIDNCPPNLGLTEADIQPQLNRRRPGQSAITTPRNE
AN5731.1  	MSTWGDYFRVTT2YGESHCRSVGCIVDGCPPGMELTEDDIQPQMTRRRPGQSALTTPRNE
          	**::*  *****  **** :**.**:*. **.: *** ****::.******* :****:*

MG03281.1 	EDKVEIQSGTEFGITLGTPIGMRVMNKDQRPKD~YGNSTMDRFPRPSHADWTYLEKYGVK
NCU05420.1	KDRVIIQSGTEFGVTLGTPIGMLVMNEDQRPKD1YGNKTMDIYPRPSHADWTYLEKYGVK
FG01643.1 	KDRVTIHSGTEDGVTLGTPILLTVPNEDQRPKD~YGNKTIDLYPRPSHADWTYLEKYGTK
AN5731.1  	KDRVEIQSGTEFGVTLGTPIAMVVRNEDQRPKD~YGNKTMDMYPRPSHADFTYLEKYGVK
          	:*:* *:**** *:****** : * *:****** ***.*:* :*******:*******.*

MG03281.1 	ASSGGGRSSARETIG~RVAAGAIAEKYLKLAYGVEIVAFVSSVGNQFLFPPTAEHPTPCT
NCU05420.1	ASSGGGRSSARETIG~RVAAGAIAEKYLKLAYGVEIVAFVSSVGSEHLFPPTAEHPSPST
FG01643.1 	ASSGGGRSSARETIA~RVAAGAVAEKWLKEAYGIEIVAFVTSVGNIKLFG---DSDSMSA
AN5731.1  	ASSGGGRSSARETIG1RVAAGAIAEKYLRLSHGVEIVAFVSSVGNEHLFPPTPEHPSPAT
          	**************. ******:***:*: ::*:******:***.  ** .:.:  : .:

MG03281.1 	NPEFLSLLQTIDRKQVDSHLPVRCPDAEASAKMRDYIAGFKDRNDSIGGTVHCVIRNAPS
NCU05420.1	NPEFLKLVNSITRETVDSFLPVRCPDAEANKRMEDLITKFRDNHDSIGGTVTCVIRNVPS
FG01643.1 	DPAFLSLAERITRQEVDDNLPVRCPDDAYGRAMEARIAELRDAHDSTGGTVTCVIRNAPS
AN5731.1  	NPEFLSLIEKIDRATVDAHAPTRCPNEAAAARMTKVIEHFRDNSDSIGGTVTCVIRNVPV
          	:* **.* : * *  **   *.***:      *   *  ::*  ** **** *****.* 

MG03281.1 	GLGEPCFDKLEAKLGHAMLSIPATKGFEIGSGFAGCQLPGSIHNDPFIKAEEAATS---A
NCU05420.1	GLGEPAFDKLEAMLAHAMLSIPATKGFEVGSGFGGCEVPGSIHNDPFVSAENTEIPPSVA
FG01643.1 	GLGEPCFDKLQATLAHAMMSIPAVKAFELGSGFQGAEMTGSKHNDPFVPAP-------AL
AN5731.1  	GLGEPCFDKLEAQLAHAMLSIPATKGFEIGSGFGGCEVPGSIHNDPFVASE--------V
          	*****.****:* *.***:****.*.**:**** *.::.** *****: :          

MG03281.1 	QNGAQHFGQSRPKLTTKTNFSGGIQGGISNGAPIYFSVAFKPPATIGQEQKTATYDGDSE
NCU05420.1	ASGAARNGIPRPKLTTKTNFSGGIQGGISNGAPIYFRVGFKPAATIGQEQTTATYDGTSE
FG01643.1 	DAASEKTGIPRSKLQTKTNHSGGIQGGISNGMPIFFKVGFKPPATISQDQTTARYDGQGE
AN5731.1  	QTQLGSQNTTKQRLVTKTNNSGGIQGGISNGASIYFRVAFKPPATIGQAQQTATYDFG-E
          	       . .: :* **** *********** .*:* *.***.***.* * ** **   *

MG03281.1 	GVLAAKGRHDPCVVPRAVPIVEAMAALVLMDAVLTQQARHTAKSLLPPLSQTLPS-QPPS
NCU05420.1	GVLAAKGRHDPSVVPRAVPIVEAMAALVIMDAVLAQQARHTAKSLLPPLKQTINSGKDTV
FG01643.1 	GILSAKGRHDPCVVPRAVPIVEGMAALVIADAIMAQHARQMATDIIKR------------
AN5731.1  	GVLEAKGRHDPCVVPRAVPIVEAMSALVVMDSLLAQYARESAKTLLPPLPKTIPT-RPTT
          	*:* *******.**********.*:***: *::::* **. *. ::    .:  :   . 

MG03281.1 	GNGVSENAAGN----
NCU05420.1	GNGVSENVQESDLAQ
FG01643.1 	---------------
AN5731.1  	GSN------------
          	...
```
